# Trail Pheromone of the Argentine Ant, *Linepithema humile* (Mayr) (Hymenoptera: Formicidae)

**DOI:** 10.1371/journal.pone.0045016

**Published:** 2012-09-20

**Authors:** Dong-Hwan Choe, David B. Villafuerte, Neil D. Tsutsui

**Affiliations:** 1 Department of Environmental Science, Policy and Management, University of California, Berkeley, California, United States of America; 2 Environmental Leadership Pathway Program, University of California, Berkeley, California, United States of America; INRA-UPMC, France

## Abstract

The Argentine ant (*Linepithema humile*) is recognized as one of the world's most damaging invasive species. One reason for the ecological dominance of introduced Argentine ant populations is their ability to dominate food and habitat resources through the rapid mobilization and recruitment of thousands of workers. More than 30 years ago, studies showed that (Z)-9-hexadecenal strongly attracted Argentine ant workers in a multi-choice olfactometer, suggesting that (Z)-9-hexadecenal might be the trail pheromone, or a component of a trail pheromone mixture. Since then, numerous studies have considered (Z)-9-hexadecenal as the key component of the Argentine ant trails. Here, we report the first chemical analyses of the trails laid by living Argentine ants and find that (Z)-9-hexadecenal is not present in a detectible quantity. Instead, two iridoids, dolichodial and iridomyrmecin, appear to be the primary chemical constituents of the trails. Laboratory choice tests confirmed that Argentine ants were attracted to artificial trails comprised of these two chemicals significantly more often than control trails. Although (Z)-9-hexadecenal was not detected in natural trails, supplementation of artificial dolichodial+iridomyrmecin trails with an extremely low concentraion of (Z)-9-hexadecenal did increase the efficacy of the trail-following behavior. In stark contrast with previous dogma, our study suggests that dolichodial and iridomyrmecin are major components of the Argentine ant trail pheromone. (Z)-9-hexadecenal may act in an additive manner with these iridoids, but it does not occur in detectable quantities in Argentine ant recruitment trails.

## Introduction

The ability of social insects to coordinate individual behaviors for colony-level tasks is central to their ecological dominance in most terrestrial ecosystems [1]. In the social insects, the intra-colony communication mediated by semiochemicals plays an important role in organizing collective activities, such as defense, reproduction, foraging, and nest relocation [2], [3]. The trail pheromones of ants, in particular, are known to play a critical role in foraging and nest relocation processes, by efficiently leading colony members to prospective food sources or nesting sites [4]–[7].

The Argentine ant, *Linepithema humile* (Mayr), has been spread by human commerce from its native South America to subtropical and Mediterranean-climate regions throughout the world [8], [9], competitively displacing native ants in its introduced range [10], [11]. In addition to its large colony size and occupation of spatially separated nests, the highly effective mass recruitment system of the Argentine ant has been recognized as one of the major mechanisms facilitating its interspecific competitive ability. For example, Argentine ant workers recruit their nestmates to food resources more quickly than do native competitors [12], [13]. Furthermore, following environmental disturbances, such as flooding, Argentine ant workers relocate their entire colony to suitable nest sites via mass recruitment more quickly than other native ant species [14]. Finally, during intraspecific aggressive encounters between Argentine ant supercolonies, enormous numbers of workers can be recruited to conflict zones, resulting in considerable worker mortality [15].

The trail pheromone of the Argentine ant has been the focus of numerous studies because of its significance in the species' mass recruitment behavior. Cavill et al. first isolated and characterized (Z)-9-hexadecenal from crude extracts of Argentine ants (whole body, dissected ventral gland) using a series of column chromatography and microchemical reactions [16], [17]. Based on the evidence that (Z)-9-hexadecenal was a ventral gland secretion, and that it strongly attracted Argentine ant workers in a multi-choice olfactometer, Cavill et al. concluded that (Z)-9-hexadecenal might be a component of the trail pheromone complex of the Argentine ant, but conservatively referred to it as “a general aggregation factor” [16], [17]. Since then, however, many researchers have primarily focused on (Z)-9-hexadecenal in studies of Argentine ant trail pheromones [18]–[22]. The concept of (Z)-9-hexadecenal as the key component of Argentine ant trails has become broadly adopted because the ants follow trails drawn with low concentrations of (Z)-9-hexadecenal. Furthermore, with the readily availability of the synthetic standard from commercial sources, some researchers have explored the possibility of using synthetic (Z)-9-hexadecenal to develop practical management strategies for invasive Argentine ant populations. For example, one study showed that synthetic (Z)-9-hexadecenal increased the consumption of sugar-based liquid baits by Argentine ants when it was mixed with the baits [23]. Similarly, several field studies have been conducted in Hawaii and Japan to test if synthetic (Z)-9-hexadecenal disrupts trail formation and subsequent foraging activity of Argentine ants [24]–[28].

Trail pheromones of many species of ants are likely to have multiple chemical components [29]–[34]. Some evidence suggests that Argentine ants have other important chemicals in their natural recruitment trail. For example, Cavill et al. reported that the pure synthetic (Z)-9-hexadecenal was less effective in attracting Argentine ants than the entire lipid fraction isolated from the ants [17]. Moreover, a trail drawn with a gaster extract was effective for 4 h, while the activity of synthetic (Z)-9-hexadecenal trails only lasted for 1 h [18], [20]. Van Vorhis Key and Baker reported that trail-following responses of Argentine ants to synthetic (Z)-9-hexadecenal trail were similar to their responses to the whole gaster extract trail, which contains ≈100 times less (Z)-9-hexadecenal than the synthetic one [20]. The same authors also reported that a 0.2 ng cm^−1^ rate of synthetic (Z)-9-hexadecenal was required to make artificial trail equally attractive with the whole gaster extract [21]. However, 0.2 ng cm^−1^ of (Z)-9-hexadecenal far exceeds physiologically relevant concentrations because individual Argentine ant workers are known to have only a few nanograms of (Z)-9-hexadecenal in their bodies [16], [20]. Furthermore, in recent attempts to disrupt the Argentine ant trail formation with synthetic (Z)-9-hexadecenal, researchers reported that some ants still followed their natural trails, even when high concentrations of synthetic (Z)-9-hexadecenal were released in the field [24], [26], [27].

**Figure 1:**
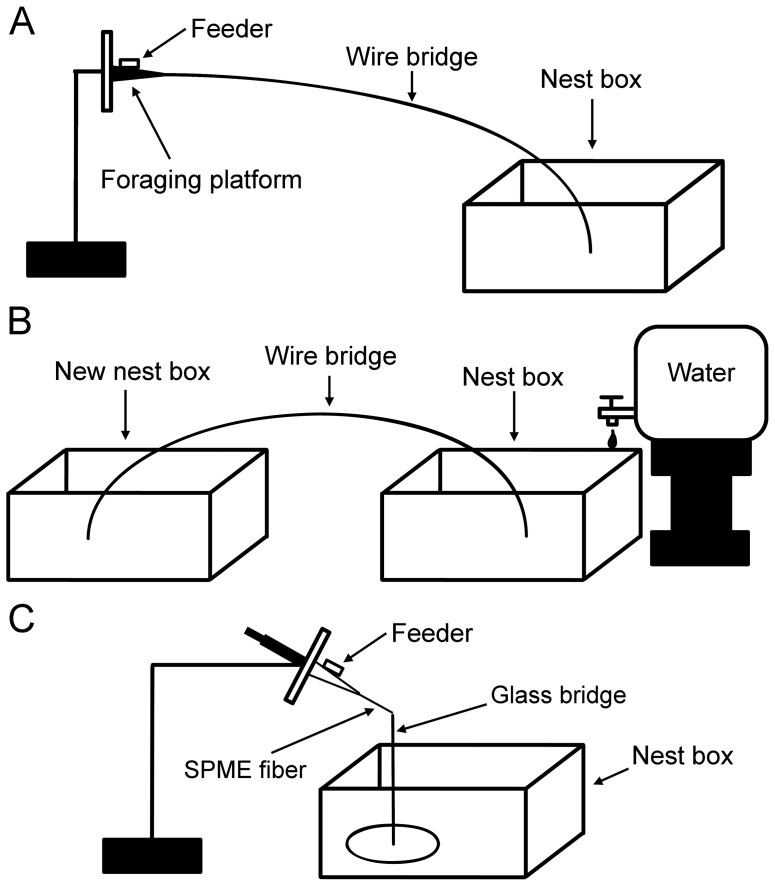
Experimental set-up for collection of trail pheromones. (A) In the foraging scenario, a sucrose solution feeder on a foraging platform was connected to a starved colony using a Teflon coated wire bridge. (B) In the flooding-induced nest relocation scenario, a water container released water drops into a nest box to induce ants to abscond into a new nest box using a Teflon coated wire bridge. (C) A SPME fiber was used to collect trail chemicals produced by foraging ants by setting it up as a short bridge leading to a sucrose solution feeder.

Here, we examined the trail substances deposited by living Argentine ants to elucidate the chemical constituents of trail pheromone. To collect the trail substances, we provided Teflon coated wire as a bridge to large laboratory colonies of the Argentine ants during two different recruitment events induced in the laboratory: foraging and nest relocation (Figure 1A and B). We also collected trail chemicals by providing solid-phase microextraction (SPME) fibers as bridges during foraging (Figure 1C). Once an active recruitment trail was established on the bridges, we either extracted the chemicals from the wire and analyzed them with coupled gas chromatography-mass spectrometry (GC-MS), or directly analyzed the chemicals adsorbed on SPME fiber with GC-MS. After identifying putative trail chemicals, we conducted bioassays to test if the compounds induced trail-following behavior. Based on our chemical analyses and behavioral bioassays, here we report two major chemical constituents of Argentine ant trail pheromone.

## Results

**Figure 2:**
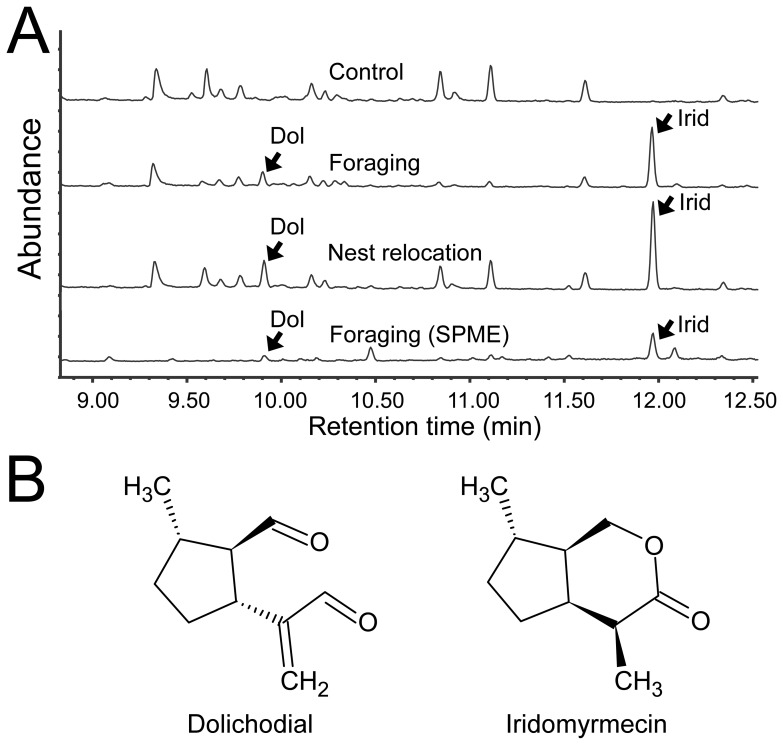
Chemical trails left by recruiting and trail-following ants during foraging and nest relocation. (A) Chromatograms of methylene chloride extracts taken from a control wire (top) and from the wires used by ants during foraging (second) and nest relocation (third). A chromatogram obtained from a SPME fiber that was used as a foraging trail is also shown (bottom). Dolichodial (Dol) and iridomyrmecin (Irid) were consistently detected on these trails (arrows). (Z)-9-hexadecenal (not detected) would have eluted with retention time of 16.03 min. (B) Chemical structures of dolichodial and iridomyrmecin, with relative configurations as shown. The absolute configurations were not determined.

GC-MS analysis revealed that two characteristic compounds were consistently present in the chemical trails deposited by Argentine ants during both foraging and nest relocation events (Figure 2A). These compounds were not found in extracts from control wires. There was no apparent difference between the chemical trails obtained from foraging and nest relocation events. Based on comparison of retention times and mass spectra to those of authentic standards, we identified the two chemicals as *trans,trans*-dolichodial [referred to as “peruphasmal” in ref. 35] and *cis,trans*-iridomyrmecin (Figure 2B). We did not find a detectible quantity of (Z)-9-hexadecenal in the trail, even though the bridges (i.e., wires and SPME fibers) were being actively used by numerous ants immediately prior to extraction.

For the colony fragments collected from Berkeley, California (Summer 2011), the concentrations of dolichodial and iridomyrmecin on the Teflon coated wire bridges (*n* = 7) were highly variable with average values of 0.1 ng cm^−1^ (range 0.01–0.17 ng cm^−1^) and 0.3 ng cm^−1^ (range 0.07–0.66 ng cm^−1^), respectively. The average amounts of dolichodial and iridomyrmecin adsorbed in 1-cm SPME fiber trail (*n* = 3) during foraing were 0.4 ng (range 0.08–0.66 ng) and 3.9 ng (range 0.43–9.1 ng), respectively. For the colony fragments collected from Riverside, California (Spring 2012), the average concentrations of dolichodial and iridomymecin on the wire bridges (*n* = 3) were 1.1 ng cm^−1^ (range 0.57–1.52 ng cm^−1^) and 3.1 ng cm^−1^ (range 0.22–5.26 ng cm^−1^), repectively. The quantitative analysis with worker ants collected from Berkeley showed that the total quantities of dolichodial, iridomyrmecin, and (Z)-9-hexadecenal in a single worker were 4.6 µg, 7.6 µg, and 13.2 ng, respectively. Based on another set of workers collected from Riverside, the total quantities of dolichodial, iridomyrmecin, and (Z)-9-hexadecenal per a single worker were estimated to be 3.8 µg, 11.7 µg, and 23.4 ng, respectively.

To test whether the mixture of dolichodial and iridomyrmecin (hereafter called MDI) attract Argentine ants, we performed Y-maze bioassays with an authentic standard of dolichodial (>99% pure) obtained from *Anisomorpha buprestoides* stick insect defensive secretion [35] and synthesized iridomyrmecin (94% pure). The compounds were dissolved in methylene chloride at a 1∶1.87 (wt∶wt, dolichodial∶iridomyrmecin) ratio to emulate the natural ratio of the two compounds found in the Argentine ants collected from Berkeley (1∶1.65, wt∶wt). A slightly higher proportion of iridomyrmecin in the standard mixture was acceptable because the ratio between those two compounds in the trail was generally skewed towards iridomyrmecin (e.g., range 1∶2–1∶14), possibly because of the relatively higher volatility of dolichodial. In the Y-maze bioassay, the standards were effective in attracting worker ants at various concentrations. More than 70% of ants chose the arms treated with the four higher concentrations (Table 1). However, authentic standard MDI, containing 6.2 pg cm^−1^ dolichodial (1.3×10^−6^-ant equivalent cm^−1^) and 11.6 pg cm^−1^ iridomyrmecin (1.5×10^−6^-ant equivalent cm^−1^), failed to attract worker ants (Table 1).

**Table 1:** Attraction of Argentine ants to different chemical trails.

Chemical	Concentration (amount/cm)	No. of replicates	% of ants choosing the treated arm (mean ± SEM)	Number of ants choosing the treated arm/total
Dolichodial/Iridomyrmecin authentic standards	6.2 ng/11.6 ng	12	76.7±7.3*	46/60
	3.1 ng/5.8 ng	12	75±3.6*	45/60
	0.62 ng/1.16 ng	24	72.5±5.1*	87/120
	62 pg/116 pg	24	70±4.5*	84/120
	6.2 pg/11.6 pg	24	45±5.5	54/120
(Z)-9-Hexadecenal	200 pg	12	93.3±2.8*	56/60
	20 pg	12	93.3±2.8*	56/60
	2 pg	12	70±6.7*	42/60
	0.2 pg	12	53.3±7.1	32/60
Control	Solvent only	12	43.3±7.3	26/60

Asterisks indicate that significantly more ants chose the treated arm over the solvent control (Chi-squared test with one-sided Dunnett-type test: α = 0.05).

Y-maze bioassays also indicated that high concentrations of synthetic (Z)-9-hexadecenal were effective at attracting worker ants, as more than 90% of ants chose the arms treated with 200 pg cm^−1^ (1.5×10^−2^-ant equivalent cm^−1^) and 20 pg cm^−1^ (1.5×10^−3^-ant equivalent cm^−1^) of (Z)-9-hexadecenal (Table 1). However, (Z)-9-hexadecenal did not attract worker ants at 0.2 pg cm^−1^ (1.5×10^−5^-ant equivalent cm^−1^) (Table 1). The threshold of (Z)-9-hexadecenal for worker attraction was lower than the threshold of MDI, indicating the worker ants are more sensitive to (Z)-9-hexadecenal than MDI at the lower concentrations.

**Figure 3:**
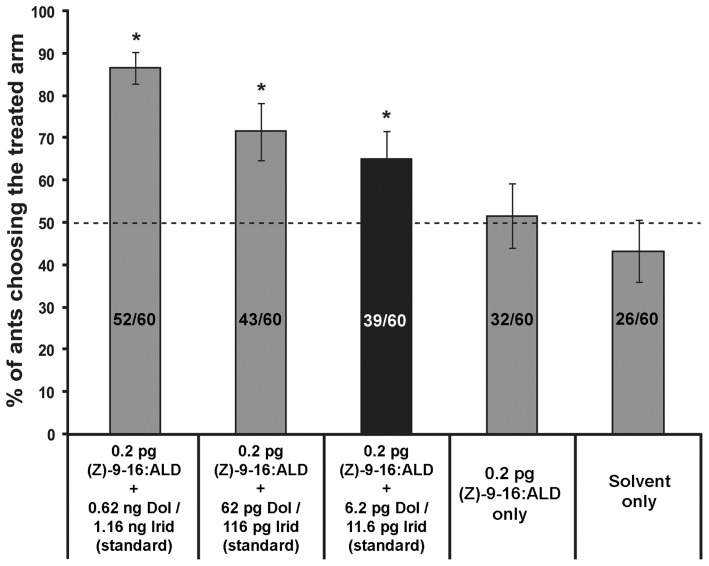
Trail choice test using dolichodial/iridomyrmecin mixture (MDI) plus subthreshod concentration of (Z)-9-hexadecenal. The height of each bar indicates the mean percentage of workers choosing the treated arm (± SEM). Bars with asterisks are significantly different from the solvent-only control (Chi-squared test with one-sided Dunnett-type test: α = 0.05). Number within a bar indicates total number of ants choosing the treated arm/total number of ants tested. The concentration shown for the chemical is the amount per cm of trail. Black bar indicate that subthreshold levels of dolichodial/iridomyrmecin were used for the particular treatment.

To determine the possible interaction between MDI and (Z)-9-hexadecenal in worker ant attraction, we performed Y-maze bioassays by simultaneously presenting various concentrations of MDI with subthreshold concentration of (Z)-9-hexadecenal (0.2 pg cm^−1^). The subthreshold concentration was defined as the maximum concentration of the chemical(s) which failed to attract workers when presented alone. We found that 65–86% ants chose the treated arm when MDI was co-presented with subthreshold rate of (Z)-9-hexadecenal (Figure 3). Most interestingly, a similar pattern was observed for subthreshold concentration of MDI: 65% of ants tested chose the arm treated with a mixture of authentic standard MDI at a subthreshold level (6. 2 pg cm^−1^ dolichodial and 11.6 pg cm^−1^ iridomyrmecin) and 0.2 pg cm^−1^ (Z)-9-hexadecenal (Figure 3, dark bar), while neither of MDI nor (Z)-9-hexadecenal attracted workers when tested individually at those concentrations (Table 1).

**Figure 4:**
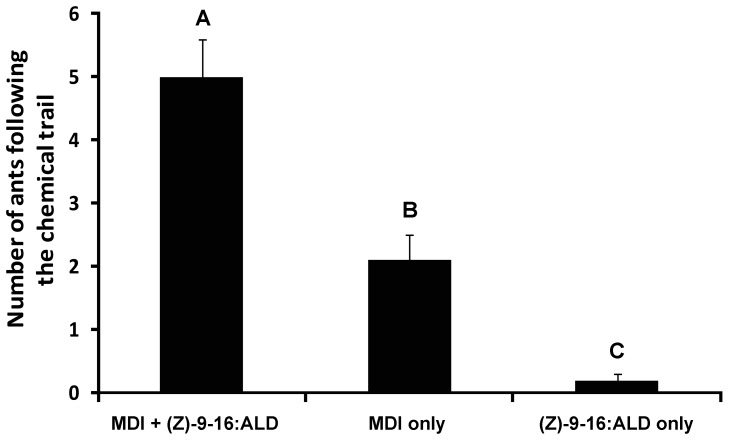
Trail-following response to dolichodial/iridomyrmecin mixture (MDI) plus (Z)-9-hexadecenal, MDI only, and (Z)-9-hexadecenal only. The height of each bar indicates the mean number of workers following the entire length of 17.3-cm trail drawn on a filter paper with the chemicals (± SEM). Concentrations of the test chemicals were 1.3 ng cm^−1^ dolichodial, 4.1 ng cm^−1^ iridomyrmecin, and 0.7 pg cm^−1^ (Z)-9-hexadecenal. Bars with different letters are significantly different (ANOVA with Tukey HSD all-pairwise comparision test: α = 0.05).

To test the effect of MDI and (Z)-9-hexadecenal in the trailing response (i.e., movement along a trail), we conducted biassays with 17.3-cm long S-shaped trails drawn on rectangular filter paper pieces (Figure S1). We tested following concentrations: 1.3 ng cm^−1^ dolichodial, 4.1 ng cm^−1^ iridomyrmecin, and 0.7 pg cm^−1^ (Z)-9-hexadecenal. The concentration for MDI was considered “physiologically relevant” based on the quantification studies with the workers collected from Riverside. Based on the total numbers of ants sucessfully following the entire trail, we found that MDI+(Z)-9-hexadenceanl trails elicited the highest trail-following responses (5.0±0.6 ants, mean ± SEM) relative to MDI only (2.1±0.4 ants) and (Z)-9-hexadecenal only (0.2±0.1 ants) trails (ANOVA with Tukey HSD all-pairwise comparision test; *F* = 37.7, df = 2, *P*<0.0001) (Figure 4).

## Discussion

Since the first identification of the trail chemicals in a leaf cutting ant, *Atta texana* [36], the study of trail pheromones has been carried out in numerous species of ants [3], [37], [38]. Because individual ants often secrete trail pheromones in subnanogram quantities [29], [30], many studies have followed an indirect approach: examination of the chemicals stored in the internal glands. This approach typically includes following three steps: (1) identification of the putative glandular source by performing bioassays with extracts of whole body, gaster, and/or dissected glands, (2) purification and identification of active chemical constituents from the crude extracts, and (3) confirmation of the pheromone's chemical identity by performing bioassays with synthesized standards and their mixtures [16], [31], [39]. Even when the glandular source of trail pherome is unknown, this standard procedure allows researchers to quickly screen various kinds of exocrine gland products for their possible activity as trail substances. However, identifying the glandular source for trail-following activity and analyzing extracts of the dissected glands usually result in fewer bioactive compounds which are likely to contribute to trail-following. Once the investigator understands the chemical properties of the target compounds, the whole body or gaster can be extracted to obtain relatively large quantity of chemicals to work with, which is often necessary for detailed chemical identification processes (e.g., derivatization).

However, this approach has two limitations. First, one cannot assume that the chemicals in the extracts, either from whole gaster or dissected glands, are the ones that are produced and deposited on the substrate by recruiting ants. Second, when several unrelated chemicals from different glands are involved in the trail-following process, it is difficult to pinpoint the optimum ratio of the individual constituents, unless various permutations at different concentrations of chemicals are tested to achieve the behavioral responses elicited by natural trails.

We found that the recruitment trails of Argentine ants, during both foraging and nest relocation, contain dolichodial and iridomyrmecin. Bioassays with standard dolichodial and iridomyrmecin confirmed that this mixture attracted workers and induced trail-following behavior. Dolichodial and iridomyrmecin are produced and stored in the pygidial gland of Argentine ants [40], [41]. A nearly pure mixture of dolichodial and iridomymecin can be “milked” from the pygidial gland by gently pressing the gasters of chilled workers with fine forceps (D.-H. Choe, unpublished data). Historically, the pygidial glands of dolichoderine ants have been believed to produce defensive secretions that provoke the alarm response, or chemicals with antibiotic/insecticidal effects [39], [41]–[48]. However, in some cases, the pygidial gland secretions of dolichoderine ants appear to play a role in other types of communication. For example, *Tapinoma simrothi* displayed a trail-following response to a pygidial gland secretion fraction that contained iridodials and iridomyrmecin [49]. Pygidial gland products of the Argentine ant, dolichodial and iridomyrmecin, are also known to play an important role in inhibiting necrophoresis in the workers [50].

The concentrations of dolichodial and iridomyrmecin on recruitment trails were variable, even though we collected and analyzed the chemicals while many ants were actively using the trails. This variation might be due to physical nature of dolichodial and iridomyrmecin: previous laboratory studies indicated that small amounts of the dolichodial/iridomyrmecin mixture (i.e., one ant-equivalent obtained via solvent extraction) dissipate from surfaces within 40 minutes under ambient conditions [50]. The ephemeral nature of dolichodial and iridomyrmecin deposits will produce trails that differ with age and length. This could provide trail-following ants with valuable information, and allow them to distinguish among different recruitment trails. In a complex network of foraging trails, for example, foragers might be able to distinguish the routes that lead to the most recently productive food sources [37].

We did not detect (Z)-9-hexadecenal in the recruitment trails of Argentine ants. However, we do not completely dismiss the notion that Argentine ants could also use (Z)-9-hexadecenal for recruitment for several reasons. First, it is possible that recruiting ants may deposit (Z)-9-hexadecenal in amounts below the detection limit of our current methods. A laboratory test with synthetic (Z)-9-hexadecenal indicated that ≈0.1 ng was the limit of detection with the GC-MS (D.-H. Choe, unpublished data). Thus, (Z)-9-hexadecenal existing on the trail with a rate of 20 pg cm^−1^or less would not be detected with our current methods (i.e., 50 cm×20 pg cm^−1^×10^−1^ = 100 pg = 0.1 ng). However, we could not detect (Z)-9-hexadecenal even when we analyzed an entire extract obtained from a 350-cm foraging trail (Teflon coated wire), suggesting that concentration of the (Z)-9-hexadecenal in the trail (if it is present) could not exceed 0.3 pg cm^−1^ (i.e., 0.1 ng/350 cm = 0.0003 ng cm^−1^ = 0.3 pg cm^−1^) (D.-H. Choe, unpublished data). Second, it is possible that (Z)-9-hexadecenal is not deposited on the substrate, but instead, is released by recruiting ants into the headspace, as a volatile. Because Argentine ants exhibit positive anemotaxis to airborne (Z)-9-hexadecenal [19], it is possible that they can follow an “airborne trail” of (Z)-9-hexadecenal. In a similar way, *Pachycondyla analis* (Latreille) [ = *Megaponera foetens* (Fabr.)] was suspected to release a recruitment pheromone from its pygidial gland into the air, stimulating other nestmates to follow behind the odor plume, which emanates from recruiting ants running in front [51]. Third, it is possible that Argentine ant workers utilize (Z)-9-hexadecenal only inside or around the nest to stimulate recruitment, but not on the recruitment trail. Similar examples can be found in ponerine ants, in which poison gland secretions contain longer-lasting orientation cues, while pygidial gland secretions have strong stimulatory effects on workers in the nest to lead them out [33], [51]. This “recruitment activation” process appears to exist in Argentine ants because, after encountering fed workers, previously inactive ants at the nest entrance are likely to follow trails more continuously than ants lacking such encounters [52].

We found that mixtures of dolichodial, iridomyrmecin, and (Z)-9-hexadecenal induced the maximum trail-following behavior. This combined effect might explain, at least in part, why whole gaster extracts and synthetic (Z)-9-hexadecenal were different in their efficacies as a trail. Because the total amounts of dolichodial and iridomyrmecin stored in a single worker are several hundred times higher than that of (Z)-9-hexadecenal, the extraction of whole ants or gasters with an organic solvent will likely yield a large amount of dolichodial and iridomyrmecin, along with a trace amount of (Z)-9-hexadecenal. We speculate that the presence of dolichodial and iridomyrmecin in gaster extracts was responsible for the superior efficacy and longevity of gaster extract trails relative to synthetic (Z)-9-hexadecenal trails, which have been previously reported. Our results also suggest that Argentine ant workers might be able to distinguish their natural recruitment trails, even with an excessive amount of synthetic (Z)-9-hexadecenal in the environment. The unique presence of dolichodial and iridomyrmecin in their natural trails might be at least partially responsible for some level of continued trail formation and recruitment activity reported in several trail disruption trials using synthetic (Z)-9-hexadecenal. The possibility of incorporating the dolichodial and iridomyrmecin in the ant control programs as trail disruptors warrant further study.

The mechanism of secretion and deposition of dolichodial and iridomyrmecin by Argentine ant workers also requires further investigation. The opening of the pygidial gland in Argentine ant worker is located on the posterior end of the gaster between the 6th and 7th abdominal tergites [2], [53]. During recruitment, Argentine ant workers typically dab their gasters on the substrate periodically [52]. During this action, a small amount of dolichodial and iridomyrmecin might be released from the pygidial gland and deposited on the substrate as a form of discontinuous line or a series of spots. Another possibility is a “passive” deposition of dolichodial and iridomyrmecin; The chemicals might be spread to other parts of their body (e.g., legs) by self grooming and adsorbed into the cutiurlar lipids, and subsequently transfered onto the trail when the ants walk on a substrate. This possibility is supported by the fact that trace amounts of iridomyrmecin have been detected in the head and thorax, even though these chemicals are exclusively produced in the gaster [41].

In conclusion, our study provides the first evidence that two pygidial gland chemicals are important constituents of recruitment trails of Argentine ant for foraging and nest relocation, and also validates a direct approach for the study of ant trail pheromones generally. The compositional variations of two known components of trail pheromone of pharaoh ant (*Monomorium pharaonis*) were recently investigated by extracting the subststrate on which the ants had walked [37]. However, to our knowledge, no ant species has ever been examined with this approach to discover unknown constituents of the trail pheromone. Given the novelty and importance of the findings that we report here, we anticipate that the same approach will yield new insights into the chemical and behavioral ecology of other ant species.

## Materials and Methods

### Ants

Argentine ant colony fragments were collected in Berkeley (2011 Summer) and Riverside (2012 Spring), California, United States, by digging up the nest from the ground. No specific permits were required for the described studies. In the laboratory, the colony was divided into several smaller experimental colonies, and each was placed in a plastic box (20×33×11.5 cm) with soil. The inner walls of the boxes were lined with Fluon to prevent ants from escaping. The experimental colonies contained queens, brood, and workers at the time of the experiments. All colonies were maintained at 21–25°C on a natural light cycle, and provided with 25% (wt/vol) sucrose solution, scrambled eggs supplemented with yeast powder and freeze-killed crickets and cockroaches (*Periplaneta americana*) two or three times a week.

### Collection of chemical trail

For the Argentine ant colony fragments collected in Berkeley, we used Teflon coated wire (50 cm×1.5 mm outer diameter Teflon tubing with metal wire inserted in it) as a substrate to collect trail chemicals in two different recruitment scenarios: foraging (*n* = 2) and nest relocation upon flooding (*n* = 5). We also collected foraging trail chemicals (*n* = 3) with solid-phase microextraction (SPME) samplers [Supelco Inc., 100-µm polydimethylsiloxane (PDMS) or 65-µm polydimethylsiloxane/divinylbenzene (PDMS/DVB)] by allowing ants to recruit their nestmates via an exposed SPME fiber bridge (1 cm in length). For the Argentine ant colonies collected in Riverside, we used Teflon coated wire (70 cm×2 mm outer diameter) as a substrate to collect trail chemicals in foraging scenario only (*n* = 3). For the foraging scenario, we starved a colony for at least three days, then workers were allowed to forage to a sucrose solution feeder by temporarily connecting the colony box with the foraging platform with the Teflon coated wire. For the SPME fiber method, we provided the test colony a glass bridge attached to the botton of colony box, allowing ants to climb up to the tip, and the tip of the glass bridge was temporarily connected to the foraging platform with a bridge of SPME fiber. For the nest relocation scenario, we slowly flooded a colony by dripping water into the colony box at a rate of one drop per second. Once evacuation began, we provided a Teflon coated wire bridge to permit access to a new nest box with dry soil. In both scenarios, the ants showed active recruitment on the wire within 30 min. The wire was carefully removed from the setting while many ants were still using it, and the entire surface of the wire was extracted with 0.5 ml of methylene chloride (trail extract). In the SPME experiment, the SPME sampler was carefully removed from the apparatus and directly analyzed by coupled gas chromatography-mass spectrometry (GC-MS). As a control, we extracted a clean Teflon coated wire that was prepared in the identical way, but not used by ants (*n* = 1).

### Chemical analysis

The trail extract was concentrated down to ≈10 µl under N_2_ flow, and 1 µl was analyzed by GC-MS. For GC-MS, electron impact mass spectra (70 eV) were taken with an Agilent 5975C mass selective detector interfaced to a Agilent 7890A gas chromatograph fitted with an DB-5 column (30 m×0.32 mm inner diameter, Agilent Technologies). Extracts were injected in splitless mode, with a temperature program of 50°C for 1 min and then 10°C min^−1^ to 300°C with 15-min hold. The temperature of injector and transfer line was 280°C. For the SPME, we set the GC injector temperature as 250°C, but otherwise the GC setting was identical. Some quantitative analyses were also conducted with a Agilent 7890 gas chromatograph equipped with a DB-5 column (30 m×0.25 mm inner diameter, Agilent Technologies) and a FID. For both GC-MS and GC analyses, helium was used as the carrier gas. Compounds in the extracts were identified by comparison of retention times and mass spectra with those of authentic standards of natural or synthetic origin. The relative configurations of dolichodial [40] and iridomyrmecin [54] produced by Argentine ants have been unequivocally determined by previous researchers, but the absolute configurations remain unknown.

We estimated the concentrations of dolichodial and iridomyrmecin on the trails using external standards of known concentrations. We also estimated the total quantities of dolichodial, iridomrymecin, and (Z)-9-hexadecenal in a single worker ant by extracting 10 workers homogenized in 200-µl methylene chloride. One or 0.5 µl (0.05- or 0.025-ant equivalent) of the crude extract was analyzed by GC or GC-MS, and the quantities of chemicals were determined using external standards.

### Behavioral bioassays

A Y-maze bioassay was developed to determine orientation choice of ants in response to candidate trail pheromone chemicals. For this study, colony fragments collected in Berkeley were used. The Y-maze (with three arms 120° apart, 4.5 cm in length, and 5 mm in width) was cut from a filter paper disk (90 mm in diameter). One arm served as the point of introduction, and the other two served as treatment and control arms. The treatment arm was treated with test chemicals dissolved in methylene chloride (3 µl) by evenly spotting the preparation over the arm with disposable glass pipettes. The control arm received an equal amount of solvent only. The introduction arm was treated with both test chemicals and solvent. For the control, all arms were treated with solvent only. To minimize the possible loss of trail chemicals due to volatilization during the solvent evaporation, we always treated control arm side first before treating the treatment arm side. We allowed the solvent to evaporate for 30 sec, and placed one treated Y-maze in the ant colony box by leaning it against a wall. Because the introduction arm was the only part contacting the soil surface, ants typically traveled upward upon encountering the introduction arm, and subsequently made a choice at the junction of the three arms. As soon as the ants reached the top of either choice arm, we removed them to prevent recruitment of other ants. We recorded the choices of five ants per Y-maze, and an individual ant was used only once. We repeated this bioassay 12 or 24 times for each chemical trail treatment (total 60 or 120 ants) in a blind manner (i.e., the examiner did not know which arm was treated with test chemicals). The data (i.e., proportion of ants choosing the treatment arm) were analyzed by a Chi-squared test. If the null hypothesis was rejected, the comparison of a control to each other treatment were conducted with one-sided Dunnett-type test [55].

To determine the effect of candidate chemicals on continous trailing, we conducted a trail-following bioassay with an S-shaped artificial trail (17.3 cm in length) drawn on a rectangular piece of filter paper (9×17 cm). For this study, a colony fragment collected from Riverside was used. The test chemicals dissolved in methylene chloride (total 12 µl) were applied by evenly spotting the preparation over a pencil-drawn guideline with disposable glass pipettes. Based on our preliminary observations, the pencil lines alone did not induce trail-following. After allowing 30 sec for the solvent to evaporate, the filter paper with artificial trail was placed in the foraging area of an large Argentine ant colony box (86×42×14 cm; containing 30–50 queens, brood, and 3,000–5,000 workers nesting in soil). Foraging workers on the soil surface readily discovered the filter paper, climbed on it, and encountered the trail. Once an ant reached the trail, we started videotaping the filter paper for 2 min until the trial was terminated. Based on the video images taken, the number of ants following the entire length of chemical trail was counted. Different chemicals were alternated between trials to insure all treatments were tested under similar activity level of the test colony. Each filter paper with trail was used only once, and we replicated this bioassay 11 times for each chemical trail treatment. The data (i.e., number of ants that followed the entire trail during the first 2 min) were square root transformed [55], and tested using ANOVA followed by Tukey HSD all-pairwise comparision test.

### Chemicals

Authentic standards of *trans,trans*-dolichodial (from *Anisomorpha buprestoides* stick insect defensive secretion, referred to as “peruphasmal” in ref. 35) and synthtic *cis,trans*-iridomyrmecin were obtained from A.T. Dossey (Gainesville, Florida) and K.R. Chauhan (USDA-ARS, Beltsville, Maryland), respectively. (Z)-9-hexadecenal was obtained from Bedoukian Research Inc. (Danbury, Connecticut). These authentic standards of dolichodial, iridomyrmecin, and (Z)-9-hexadecenal were examined with GC-MS in prior to bioassays, showing purities of >99, 94, and 97%, respectively.

## Supporting Information

Figure S1 **Trail-following bioassay setup to test the continuous trailing response of workers.** Dolichodial/iridomyrmecin mixture (MDI) plus (Z)-9-hexadecenal, MDI only, and (Z)-9-hexadecenal only were tested on the trail. The S-shaped curve was drawn on a retangular filter paper (9×17 cm) with a circlular template of 5.5-cm diameter. The side of the filter paper was cut in a triagular shape, so that the encounter of ants with the chemical trails was fasciliated. The total number of workers which successufuly followed the entire trail (red arrows) were recorded for 2 min.
(TIF)
