# Supplementary figures and images for: Trail Pheromone of the Argentine Ant, Linepithema humile (Mayr) (Hymenoptera: Formicidae)
Source: PLoS One. 2012 Sep 20;7(9):e45016. doi: 10.1371/journal.pone.0045016 (PMC3447822; doi:10.1371/journal.pone.0045016)

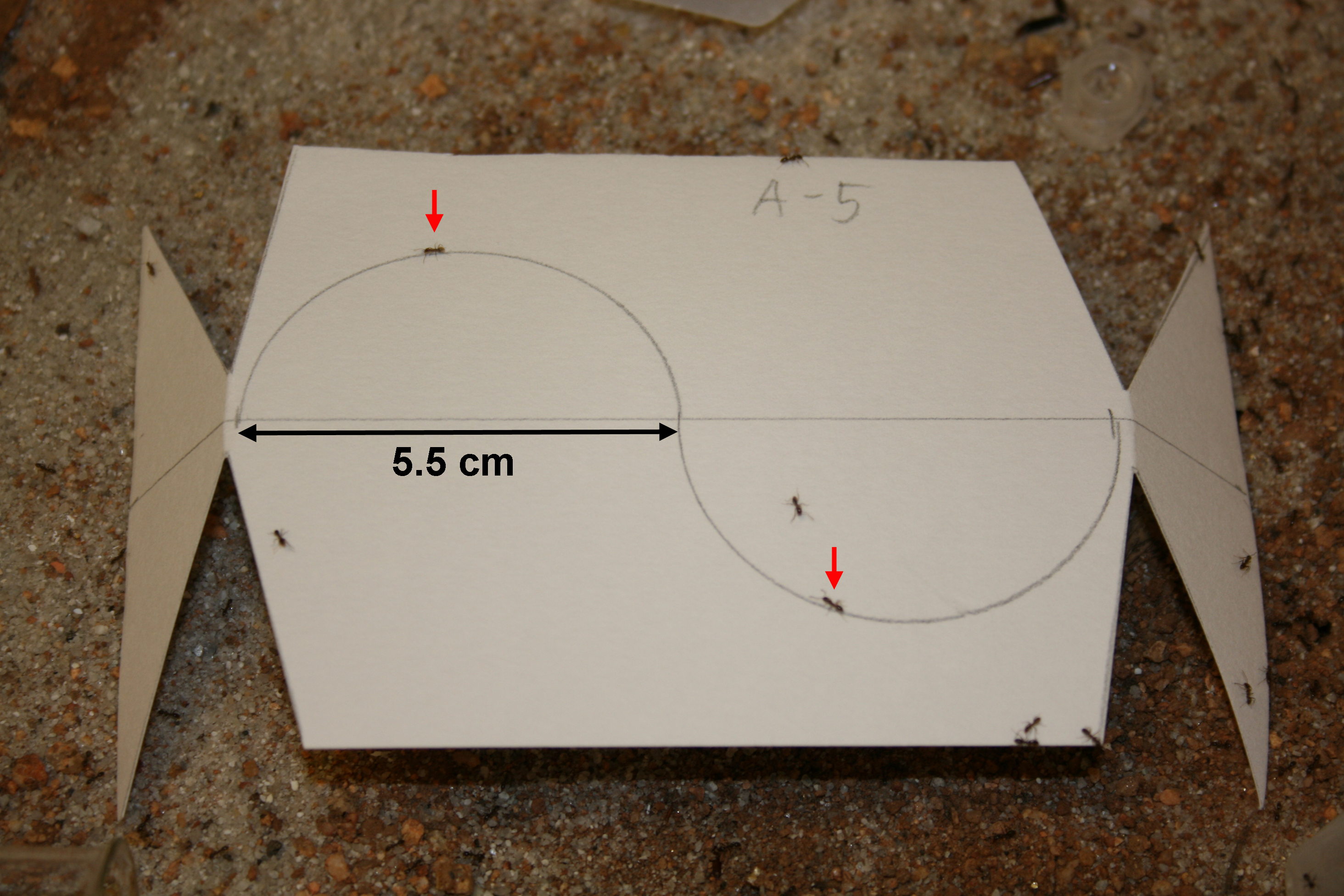

Supplement: Figure S1 — Trail-following bioassay setup to test the continuous trailing response of workers. Dolichodial/iridomyrmecin mixture (MDI) plus (Z)-9-hexadecenal, MDI only, and (Z)-9-hexadecenal only were tested on the trail. The S-shaped curve was drawn on a retangular filter paper (9×17 cm) with a circlular template of 5.5-cm diameter. The side of the filter paper was cut in a triagular shape, so that the encounter of ants with the chemical trails was fasciliated. The total number of workers which successufuly followed the entire trail (red arrows) were recorded for 2 min. (TIF) [file pone.0045016.s001.tif]
